# Beyond Linear Growth: Adaptive Human Intelligence, Emotional Dynamics, and Willingness to Write in Artificial Intelligence-Mediated Informal Digital Learning of English (AI-IDLE)

**DOI:** 10.3390/jintelligence14100259

**Published:** 2026-10-13

**Authors:** Chenghao Wang, Lanfang Sun, Bin Zou, Yunyan Zhang

**Affiliations:** 1 Department of Applied Linguistics and Language Sciences, Xi’an Jiaotong-Liverpool University, Suzhou 215123, China; chenghao.wang21@student.xjtlu.edu.cn; 2 School of Art, University of Liverpool, Liverpool L69 3BX, UK; 3 Artificial Intelligence and Digital Competency Education Centre, The Education University of Hong Kong, Tai Po, Hong Kong, China; laforsun@connect.hku.hk; 4 Academic Literacies Centre, Xi’an Jiaotong-Liverpool University, Suzhou 215123, China

**Keywords:** human intelligence, willingness to write, Complex Dynamic Systems Theory, technology-related enjoyment, technology-related anxiety, AI-IDLE

## Abstract

The development of generative artificial intelligence (GenAI) is expanding AI-mediated informal digital learning of English (AI-IDLE) from predominantly receptive and conversational activities to cognitively demanding productive tasks such as academic writing. Yet little is known about how learners’ motivation and technology-related emotions develop over time, or how sustained interaction with GenAI may engage human capacities for evaluation and adaptation. Grounded in Complex Dynamic Systems Theory (CDST), this study investigated the longitudinal development of willingness to write (WTW), technology-related enjoyment (ENY), and technology-related anxiety (ANX) among Chinese undergraduates learning English as a Foreign Language (EFL) in teacher-supported AI-IDLE embedded within an 11-week English for Academic Purposes writing course. Twenty-six participants with complete records across 11 measurement occasions were included in the longitudinal analysis. Trajectory analysis, change-point analysis, and exploratory moving-window correlation analysis were combined with thematic analysis of learners’ reflections. WTW followed a nonlinear trajectory, declining during the middle period before a potential late-stage transition and subsequent increase, whereas ENY remained comparatively stable and ANX generally declined. The relationships of WTW with ENY and ANX also varied over time. Learners’ reflections revealed increasing recognition of human–AI complementarity and more selective, critical, and strategic engagement with GenAI. From a human-intelligence perspective, these patterns illustrate adaptive and metacognitive capacities involved in evaluating AI-generated information, adjusting strategies, and coordinating human and artificial resources. Rather than treating WTW or emotions as measures of intelligence, the findings position AI-IDLE as a context in which such human intellectual capacities may be exercised and expressed.

## 1. Introduction

Generative AI (GenAI) chatbots have entered university writing faster than research has tracked their psychological effects on learners (F. Sun et al., 2026; L. Sun et al., 2026). Their development is also expanding the scope of AI-mediated informal digital learning of English (AI-IDLE), extending it beyond predominantly receptive and conversational activities to cognitively demanding productive tasks such as academic writing (Guan & Gu, 2026; Qu et al., 2026). Learners may use GenAI outside the classroom to generate ideas, clarify academic concepts, evaluate language choices, or support drafting and revision. Importantly, such engagement can coexist with formal instruction. Recent research suggests that teachers may provide pedagogical support or structured guidance for out-of-class digital learning while learners retain agency over their subsequent digital engagement (Kusuma et al., 2024; Guo et al., 2026; Lee et al., 2026). Writing tasks may therefore be structured within the curriculum and GenAI tools introduced and demonstrated by teachers, while learners retain discretion over whether, when, and how to use these tools outside class. Such teacher-supported AI-IDLE illustrates how formal instructional structure can coexist with learner-directed out-of-class digital engagement (Guo et al., 2026; Lee et al., 2026).

Existing research has provided growing evidence concerning the role of GenAI in second language (L2) writing, including its implications for writing performance, feedback, technology acceptance, and learner experience (Liang et al., 2024; G. L. Liu et al., 2025; Qu et al., 2026; Zou et al., 2025). Less is known, however, about how learners’ writing-specific willingness and affect develop across repeated episodes of GenAI-supported writing. Within a single course, learners may accumulate experience with both increasingly demanding academic writing activities and the affordances and limitations of GenAI. Their engagement with writing may therefore fluctuate rather than develop in a simple linear direction. Cross-sectional surveys and conventional pre–post designs provide only limited insight into such temporal variation.

Willingness to write (WTW) offers a useful lens through which to examine this developmental process (Kaivanpanah et al., 2019; Zou et al., 2025). Unlike a single act of speaking up in class, sustained academic writing requires learners to engage repeatedly in processes such as planning, drafting, feedback, and revision (Feng et al., 2025; Zou et al., 2025), and there is little reason to assume that their readiness to engage remains constant throughout these processes. In teacher-supported AI-IDLE, this dynamic may be particularly complex because learners respond to progressively structured writing demands while simultaneously exercising discretion over their out-of-class GenAI use. WTW may therefore vary as writing tasks, learner experience, and patterns of GenAI engagement develop over time. Whether and how such willingness fluctuates cannot be adequately captured by a survey administered at a single time point or solely before and after a course.

The picture is complicated further by the fact that GenAI is a source of affective experience as well as writing support. Learners may value its immediacy and accessibility while simultaneously encountering generic, inaccurate, or insufficiently task-specific suggestions (L. Sun et al., 2026; C. Wang et al., 2026; Zou et al., 2025). Technology-related enjoyment and anxiety need not develop in opposite directions or in parallel (Pawlak et al., 2025; Shi et al., 2026), and their relationships with WTW may likewise vary across repeated learning experiences. Yet the cross-sectional and conventional pre–post designs common in this literature (Huang et al., 2021; C. Wang et al., 2026; Zou et al., 2025) provide limited insight into when such fluctuations occur or how the relationships among WTW and affective experiences vary over time. A longitudinal perspective is therefore needed to examine these constructs as dynamic and context-sensitive processes rather than static learner characteristics.

In AI-mediated learning, human intelligence is relevant not simply because learners interact with an intelligent system, but because such interaction requires learners to evaluate generated information, recognise technological limitations, adapt strategies to changing task demands, and determine how human and artificial resources should be coordinated (C. Wang et al., 2026; Zou et al., 2025). These evaluative, adaptive, and metacognitive capacities become particularly salient in sustained academic writing, where learners must repeatedly decide whether and how AI-generated support should be incorporated into their own intellectual work (G. L. Liu et al., 2025; Zou et al., 2025). From this perspective, examining how learners’ willingness and technology-related emotions develop during AI-IDLE is not only a question about writing engagement but also a question about the adaptive human capacities exercised in AI-rich learning environments.

The present study addresses this need through the lens of Complex Dynamic Systems Theory (CDST), which conceptualises motivation and emotion as dynamic, interconnected, and sensitive to ongoing interactions between learners and their learning environments (De Bot et al., 2007; Larsen-Freeman, 1997). We tracked the WTW, technology-related enjoyment (ENY), and technology-related anxiety (ANX) of 26 Chinese undergraduates learning English as a foreign language (EFL) across 11 measurement occasions corresponding to an 11-week English for Academic Purposes (EAP) course. The academic writing activities were progressively structured through formal instruction, and a customised GenAI chatbot was introduced and demonstrated by the instructor during the first class. Thereafter, learners could voluntarily decide whether, when, and how to use the chatbot to support their out-of-class academic writing. The study therefore examines teacher-supported AI-IDLE embedded within formal EAP instruction, conceptualised more specifically as learner-directed, out-of-class engagement with GenAI in support of formally instructed English writing. Trajectory analysis, change-point analysis (CPA), and exploratory moving-window correlation (MWC) analysis were combined with learners’ written reflections to examine the longitudinal trajectories of WTW, ENY, and ANX, potential transitions in WTW, temporal variation in their within-person covariation, and learners’ experiences surrounding these patterns.

## 2. Literature Review

### 2.1. AI-IDLE and AI-IDLE Writing

Informal digital learning of English (IDLE) refers broadly to learners’ self-directed engagement with digital resources beyond the formal classroom through which they encounter, use, and produce English (Lee, 2022). Rather than being defined by a particular technology, IDLE has traditionally been characterised by learner agency and substantial autonomy over what, when, and how digital resources are used for language learning. A substantial body of research has linked IDLE participation to language proficiency, willingness to communicate, and a range of affective outcomes, while also suggesting that the diversity of learners’ digital practices may be as important as the amount of engagement itself (Lee, 2022; Lee & Hsieh, 2019; G. L. Liu & Hossain, 2026; Y. Wang & Guo, 2026).

The emergence of GenAI has further expanded IDLE into AI-IDLE. Compared with earlier digital resources that primarily facilitated access to existing content, GenAI enables learners to generate, revise, evaluate, and discuss language through responsive interaction, thereby broadening the range of language-learning activities available outside the classroom. Research on AI-IDLE has consequently examined adoption patterns and learner experiences (G. L. Liu et al., 2025) and motivational and appraisal antecedents of engagement (X. Zhou & Liu, 2026). Studies have also identified learner-level factors associated with AI-IDLE engagement, including English learning confidence, digital competence, perceptions of GenAI, motivation, and self-efficacy (G. L. Liu & Zhao, 2026; J. Liu et al., 2026).

Importantly, learner agency in IDLE does not necessarily require the complete absence of teacher involvement. Recent research has increasingly recognised forms of IDLE in which teachers provide pedagogical structure or support while learners retain meaningful agency over their out-of-class digital engagement. Kusuma et al. (2024), for example, examined out-of-class IDLE activities supporting formal speaking instruction, while Guo et al. (2026) explicitly distinguished self-directed AI-IDLE from teacher-supported AI-IDLE. Similarly, Lee et al. (2026) examined teacher-supported IDLE for L2 writing alongside formal weekly writing instruction. This distinction is particularly relevant to academic writing because the initiation of a writing task and the initiation of digital engagement do not necessarily coincide. Academic writing may originate from coursework, assessment, or other instructional requirements (Seviour, 2015), while learners may still exercise agency over their subsequent use of digital resources outside the classroom. Thus, formal instructional structure and informal digital engagement need not be mutually exclusive; learner agency may reside in how learners engage with digital resources to support formally initiated writing tasks.

GenAI makes this distinction increasingly salient in L2 writing. With its capabilities for text generation, analysis, and language processing, GenAI can support brainstorming, outlining, content organisation, revision, feedback provision, proofreading, and assessment (Feng et al., 2025; Jeon et al., 2023; L. Sun et al., 2026). Such support is available on demand, allowing learners to decide when assistance is needed, which aspects of their writing to seek support for, how to formulate requests, and whether to accept, modify, or reject AI-generated suggestions. Research has reported that AI-supported writing can enhance linguistic proficiency and writing quality while also shaping learners’ psychological experiences, including writing confidence, self-efficacy, motivation, engagement, enjoyment, boredom, and writing anxiety (Alghamdi, 2024; Ghafouri et al., 2024; L. Sun et al., 2026). Recent evidence, however, suggests that these affective consequences are not uniformly positive. For example, F. Sun et al. (2026) found that LLM acceptance was positively associated with both enjoyment and anxiety in L2 academic writing, indicating that engagement with GenAI may activate simultaneous positive and negative emotional pathways. Similarly, Tanino (2026) showed that GenAI use reduced some dimensions of writing anxiety, such as somatic anxiety and avoidance behaviour, while increasing communication apprehension. These findings point to a more complex affective picture than a simple beneficial–detrimental distinction. Nevertheless, psychological responses to AI-assisted writing have still been examined primarily as outcomes observed at particular points in time, leaving less understood about how such experiences develop across repeated episodes of AI-supported writing.

Against this background, the present study conceptualises AI-IDLE writing as a teacher-supported form of AI-IDLE in which formal writing requirements coexist with learner-directed out-of-class GenAI engagement. The EAP writing tasks were embedded within the formal curriculum and progressively structured by the instructor and are therefore not treated as entirely learner-initiated informal activities. However, learners retained discretion over whether, when, and how to use the GenAI chatbot outside class, which aspects of their writing to seek support for, and whether to continue using it beyond immediate instructional requirements. Accordingly, AI-IDLE writing in the present study refers to learner-directed, out-of-class engagement with GenAI in support of formally instructed English writing.

Two limitations of this evidence base motivate the present study. The first concerns research design. Much of the existing evidence relies on cross-sectional surveys or conventional pre–post designs, which primarily indicate whether differences or benefits are observable at particular measurement points. Such designs provide limited insight into how learners’ motivational and affective responses develop across repeated episodes of GenAI-supported writing. Whether these responses change steadily as learners gain experience, fluctuate over time, or shift after periods of difficulty therefore remains difficult to determine. The second concerns sustained engagement. Unlike brief or receptive forms of digital engagement, extended academic writing unfolds over time and requires learners to repeatedly decide whether to initiate, persist in, or return to writing as difficulties arise. GenAI may facilitate this process by providing accessible and immediate support, but learners’ willingness to continue writing may also vary as they encounter limitations in the relevance, depth, or reliability of AI-generated assistance. Consequently, understanding AI-IDLE writing requires attention not only to what learners gain from GenAI use, but also to whether their willingness to engage in writing is sustained, declines, or recovers across repeated experiences. This temporal dimension calls for a construct that captures learners’ readiness to engage in writing and an analytical perspective capable of examining its fluctuation and change over time.

### 2.2. Willingness to Write in GenAI-Assisted Writing

Willingness to communicate (WTC) has long been conceptualised as the final psychological step before an L2 user actually engages in communication, shaped by both stable individual differences and momentary situational factors (MacIntyre et al., 1998). Although WTC research has traditionally focused on speaking, the construct has been extended to digital and computer-mediated contexts, where learners’ willingness to engage differs across in-class, out-of-class, and digital environments (Lee & Hsieh, 2019). Willingness to write (WTW) is a skill-specific extension of this logic (Kaivanpanah et al., 2019; Yang & Zhang, 2023; Zou et al., 2025). In L2 contexts, WTW broadly refers to learners’ willingness to engage in writing activities in the target language and reflects their readiness to initiate and sustain writing engagement (Kaivanpanah et al., 2019). In GenAI-assisted EFL writing, WTW has further been treated as an important affective and motivational indicator of learners’ engagement with writing and revision activities (Zou et al., 2025). Unlike spoken communication, which is typically immediate, brief, and interactionally co-constructed, writing is sustained and relatively solitary, requiring learners to remain willing to engage throughout composing, revising, and refining a text.

This sustained nature is what makes WTW important for the present purpose. Producing an academic text requires learners to develop and refine ideas over time, tolerate uncertainty about the quality of their arguments, and persist through cognitively and emotionally demanding stages (Boscolo & Hidi, 2007; Zou et al., 2025). Boscolo and Gelati (2019) noted that learners’ motivation to write may change as they engage with the demands of writing tasks. From this perspective, WTW may likewise fluctuate as learners encounter the cumulative demands of sustained composition. Since WTW is closely associated with engagement, persistence, and the effort learners invest in revision and idea development (Kaivanpanah et al., 2019; Yang & Zhang, 2023; Zou et al., 2025), its development over time may have important implications for whether learners sustain their engagement with an extended writing project. Rather than remaining stable throughout the writing process, WTW may therefore rise or fall as learners respond to the changing demands and resources encountered during sustained composition.

The emergence of GenAI introduces a new layer of support and complexity into this process. Chatbot support may sustain or raise WTW by lowering the cognitive cost of getting started, clarifying writing problems, and carrying learners through difficult stages of composition (Zou et al., 2025). It may equally generate friction: extensive AI-generated suggestions can produce information overload, and reliance on the tool may substitute for the development of learners’ own writing strategies (Jeon et al., 2023; G. L. Liu et al., 2025). During out-of-class AI-IDLE writing, learners may need to navigate these tensions without immediate instructor guidance. The relationship between GenAI support and WTW is consequently unlikely to be uniformly positive, or stable, across an extended period of writing. Yet despite growing interest in AI-supported academic writing, research has largely addressed writing performance, learners’ general perceptions, feedback quality, and technology-acceptance variables such as perceived usefulness and perceived ease of use (Liang et al., 2024; G. L. Liu et al., 2025; Zou et al., 2025), and WTW has received little attention as a motivational construct in its own right.

### 2.3. Technology-Related Enjoyment and Anxiety in GenAI-Assisted L2 Writing

The constructs of ENY and ANX in this study are deliberately distinguished from the broader constructs of foreign language enjoyment (FLE) and foreign language classroom anxiety (FLCA), which have been widely examined in second language acquisition. FLE and FLCA capture learners’ emotional responses to language learning more generally, including the classroom environment, teacher, peers, language tasks, and the target language itself (Dewaele & MacIntyre, 2014; Horwitz et al., 1986). By contrast, ENY and ANX in the present study refer specifically to learners’ emotional responses to GenAI as a writing-support technology. Drawing on technology-acceptance and technology-anxiety research (Fagan et al., 2008; Heinssen et al., 1987; Venkatesh et al., 2012; Y.-Y. Wang & Wang, 2022), ENY refers to the enjoyment or pleasure learners experience when interacting with GenAI during academic writing, whereas ANX refers to the uneasiness, tension, or apprehension associated with using GenAI to support writing. In this sense, the present study shifts attention from how technology influences language-learning emotions in general to how technology itself becomes an emotional object that can generate enjoyment, anxiety, or both during the learning process.

This technology-oriented understanding of enjoyment is grounded in technology-acceptance research. The Technology Acceptance Model (TAM) (Davis, 1989) and its later extensions (Fagan et al., 2008; Venkatesh & Davis, 2000) have long recognised that users’ evaluations of a technology are not purely functional. Later models explicitly incorporated hedonic motivation, defined as the pleasure or enjoyment derived from technology use, as an important factor shaping technology adoption and continued use alongside utilitarian beliefs such as perceived usefulness (Venkatesh et al., 2012). In language learning contexts, enjoyment in AI-mediated interaction has also been linked to learners’ willingness to continue engaging with AI-supported speaking tasks (Huang & Zou, 2024). This suggests that technology-related enjoyment may encourage sustained engagement, exploration, and persistence in GenAI-assisted writing.

Technology-related anxiety has a similarly established background in information systems research. Early work on computer anxiety conceptualised it as the apprehension or fear experienced when using computers, as reflected in instruments such as the Computer Anxiety Rating Scale developed by Heinssen et al. (1987). More recently, the increasing prevalence of AI systems has led researchers to conceptualise AI anxiety as a more specific form of technology-related anxiety, involving concerns about AI reliability, autonomy, unpredictability, and potential consequences (Y.-Y. Wang & Wang, 2022). This form of anxiety is conceptually narrower than general language anxiety because it is directed at the technological system itself rather than at language learning or communication as a whole.

In GenAI-assisted academic writing, ENY and ANX may arise from overlapping features of the same tool. Immediate feedback, 24 h availability, and the novelty of interacting with an AI writing assistant or chatbot may foster enjoyment and curiosity (Zou et al., 2025). At the same time, inaccurate output, hallucinated information, excessive or overly complex suggestions, and uncertainty about whether to trust AI-generated content may increase anxiety (Zou et al., 2025). ENY and ANX should therefore not be treated as simple opposites or assumed to move in parallel. Learners may enjoy interacting with GenAI while simultaneously feeling uncertain or anxious about the reliability and appropriateness of its suggestions (Pawlak et al., 2025; Shi et al., 2026).

The IDLE setting alters what these emotions are about. In instructed use, a teacher absorbs part of the uncertainty by indicating which suggestions are worth taking and where the tool is likely to be unreliable. In out-of-class AI-IDLE writing, such immediate guidance may not be available: learners consult the tool and assess whether its responses are adequate for their disciplinary purposes. Anxiety here is therefore less a matter of unfamiliarity with an interface than of uncertainty about one’s own capacity to evaluate what the system produces. As learners gain experience with the tool, this anxiety may change as they become better able to anticipate its strengths and limitations, although such development need not be linear.

### 2.4. A CDST Perspective on Change in AI-IDLE

CDST offers a useful framework for examining these developmental questions. CDST conceptualises language development, together with the motivational and emotional processes accompanying it, as dynamic, nonlinear, adaptive, and shaped by interactions among multiple interconnected components (De Bot et al., 2007; Larsen-Freeman, 1997). From this perspective, temporal variability is theoretically meaningful because it may reflect learners’ changing responses to tasks, tools, experiences, and learning contexts rather than simply variation around a fixed underlying state.

Applied to emotion, CDST challenges the assumption that enjoyment and anxiety are stable learner traits, treating them instead as context-sensitive states that may vary through ongoing interactions among learners, tasks, tools, and environments (Dewaele & Dewaele, 2017; Jin et al., 2026). The same learner’s enjoyment of or anxiety about an activity or technological tool may therefore change as task demands, prior experience, and interactional conditions evolve (G. L. Liu et al., 2025). CDST-informed research has documented considerable temporal variability in L2 learners’ emotions and emotion regulation and has illustrated that developmental trajectories may include periods of relative stability, fluctuation, and more pronounced change rather than following a uniformly linear pattern (Dewaele & Pavelescu, 2021; Ranjbar et al., 2025; Q. Zhou, 2024).

CDST provides a useful lens for examining AI-IDLE because learners’ engagement with GenAI may change as they gain experience with the technology and encounter different learning demands over time. Importantly, such dynamics are not limited to entirely autonomous or unscaffolded learning. As discussed in Section 2.1, teacher-supported IDLE may combine formal instructional structure with learner agency over subsequent out-of-class digital engagement (Kusuma et al., 2024; Guo et al., 2026; Lee et al., 2026). In AI-IDLE writing, learners may therefore encounter changing writing demands while simultaneously accumulating experience with the affordances and limitations of GenAI. From a CDST perspective, WTW and technology-related emotions may consequently fluctuate as these learner–task–tool–context interactions develop over time. From this perspective, intelligence in AI-mediated learning can also be considered in terms of learners’ adaptive capacity to modify their strategies and regulate their engagement in response to changing task, technological, and emotional demands. However, much existing AI-IDLE research has relied on cross-sectional designs (G. L. Liu et al., 2025; Y. Wang & Guo, 2026; C. Wang et al., 2026), while CDST-informed research has more commonly examined classroom-based language learning and general language-learning emotions (Dewaele & Pavelescu, 2021; Ranjbar et al., 2025; Q. Zhou, 2024). The temporal development of motivation and emotion during sustained AI-IDLE therefore remains comparatively underexplored.

Longitudinal research on AI-mediated language learning has begun to address this temporal dimension. Shi et al. (2026), for example, traced foreign language writing enjoyment in AI-mediated contexts using latent growth curve modelling, demonstrating the value of examining change over time. Growth-curve approaches are particularly useful for estimating systematic developmental trends specified within a particular functional form. However, complementary approaches may be useful when the research question concerns whether and when potential transitions occur without specifying their timing in advance. Examining AI-IDLE from a CDST perspective may therefore benefit from combining methods that describe overall trajectories with methods sensitive to potential changes in those trajectories and temporal variation in the relationships among co-developing variables.

Three complementary approaches are adopted in the present study. Trajectory analysis describes the overall longitudinal pattern of each variable, while CPA identifies potential locations at which the mean structure of a time series changes without requiring the timing of such changes to be specified in advance. MWC is additionally used to explore temporal variation in the within-person covariation between WTW and the two affective variables across successive time windows. Peng and Liang (2025) demonstrated the value of intensive longitudinal measurement for examining learners’ WTC in AI-mediated interaction using repeated momentary measures. Building on this longitudinal orientation, the present study focuses on academic writing, where engagement develops across successive writing activities over a period of weeks, and incorporates learners’ written reflections to contextualise their experiences surrounding the quantitative patterns.

The inclusion of MWC further reflects the relational emphasis of CDST. Development may involve not only change in individual variables but also temporal variation in how motivational and affective processes covary. Existing studies of motivation and emotion in AI-mediated learning have often examined these constructs separately or modelled their associations as relatively stable across a study period (G. L. Liu et al., 2025; Shi et al., 2026). Consequently, less is known about whether the within-person covariation of WTW with ENY and ANX varies across repeated GenAI-supported writing experiences. Given the short three-occasion windows used in the present study, however, MWC is employed as an exploratory and descriptive tool, rather than as inferential evidence of structural reorganisation.

Taken together, a CDST perspective directs attention to the trajectories, potential transitions, and time-varying covariation of WTW and technology-related emotions during sustained engagement with GenAI-supported writing. In the present study, these processes are examined within teacher-supported AI-IDLE embedded in formal EAP instruction, where progressively structured writing activities coexist with learner-directed out-of-class GenAI engagement. The study does not assume that observed changes are attributable to GenAI alone; rather, they are examined as longitudinal patterns occurring within a context in which task progression, learner development, and accumulated GenAI experience unfold concurrently. On this basis, the following research questions are addressed:

RQ1. How do learners’ WTW, ENY, and ANX develop across 11 measurement occasions during teacher-supported AI-IDLE for academic writing?

RQ2. Are there potential change points in the development of WTW, ENY, and ANX?

RQ3. How does the within-person covariation between WTW and ENY, and between WTW and ANX, vary over time?

RQ4. How do learners’ reflections contextualise the observed developmental patterns?

## 3. Methods

### 3.1. Participants and Data Collection

The study was conducted in an EAP writing course involving 127 Chinese senior undergraduate students from Humanities and Social Sciences (HSS) disciplines. Participants were recruited from students enrolled in this course, and participation in the research was entirely voluntary. At the beginning of the course, the instructor introduced a GenAI chatbot as an optional learning resource and demonstrated how it could support idea generation, drafting, revision, language refinement, and source exploration for academic writing. Rather than integrating the chatbot into compulsory classroom activities, the instructor recommended its use for completing weekly out-of-class writing tasks while leaving the decision of whether, when, and how to use the chatbot entirely to individual students. This learning arrangement reflected the principles of AI-IDLE, in which learners voluntarily engage with AI technologies beyond formal instructional requirements to support their own language learning.

The course lasted 11 weeks and aimed to develop students’ academic writing competence through progressively more demanding writing tasks, beginning with academic writing conventions and progressing to topic selection, research question formulation, literature review writing, argument development, and complete academic essays. The final week was designated for self-directed learning, during which students could independently consolidate and apply what they had learned throughout the course. Students who chose to use the chatbot were invited, but not required, to complete a brief online questionnaire and a reflective journal each week to report their WTW, technology-related emotions, and experiences of using GenAI during their independent writing process. Following each weekly class, a link to the online questionnaire and reflective journal was shared in the course WeChat group, allowing students to decide whether and when to complete them at their own convenience. Neither chatbot use nor questionnaire completion formed part of the course requirements or contributed to course assessment. Students were informed that choosing not to use the chatbot or participate in the research would have no effect on their course participation or assessment.

Longitudinal data were collected on 11 measurement occasions corresponding to the 11 weeks of the course. The GenAI chatbot was introduced and demonstrated during the first class in Week 1, after which students could voluntarily use it to support their out-of-class writing. The course was held every Monday, and participants were asked to complete the corresponding questionnaire by Friday following that week’s learning activities. Accordingly, T1 was collected after the Week 1 class and the initial introduction of the chatbot and therefore represented the first post-class measurement rather than a pre-course or pre-exposure baseline. T2–T10 corresponded to the subsequent weeks of progressively structured EAP writing activities, while T11 corresponded to the final self-directed learning week. Of the 127 students enrolled in the course, 111 completed the T1 questionnaire. The numbers of participants contributing quantitative data across T1–T11 were 111, 97, 91, 85, 76, 61, 46, 27, 27, 27, and 26, respectively. Informed consent was obtained before questionnaire completion at each measurement occasion, and participation remained voluntary throughout the longitudinal data collection. For the final longitudinal analysis, only learners with complete quantitative questionnaire records across all 11 measurement occasions were included. Completion of the open-ended reflections was not an inclusion criterion. Twenty-six learners met this requirement and were retained in the final analytic sample. The complete quantitative dataset therefore comprised 286 questionnaire records (26 participants × 11 occasions). The final analytic sample included 18 females and 8 males, aged 21–23 years.

### 3.2. Instruments

#### 3.2.1. Weekly Questionnaire

The weekly questionnaire consisted of three parts. Following the intensive longitudinal measurement approach adopted by Peng and Liang (2025), WTW, ENY, and ANX were repeatedly assessed at each measurement occasion. WTW was measured using a single-item 0–100 rating scale to capture learners’ momentary willingness to engage in GenAI-assisted academic writing. The single-item format also helped minimise participant burden associated with repeated measurement across 11 occasions. Higher scores indicated greater willingness to write.

The second part measured ENY with three five-point Likert-scale items adapted from Zou et al. (2025), such as “I find using GenAI for academic writing interesting.” Across the 11 measurement occasions, the ENY scale demonstrated acceptable to excellent internal consistency, with Cronbach’s α ranging from 0.753 to 0.950. The third part measured ANX with four five-point Likert-scale items adapted from Huang et al. (2021), such as “I feel uneasy when using GenAI for academic writing.” The ANX scale also showed good to excellent internal consistency across the 11 measurement occasions, with Cronbach’s α ranging from 0.848 to 0.973.

All questionnaire items were presented bilingually in Chinese and English. The items were translated with particular attention to preserving the original meaning of the source measures. The bilingual versions and their adaptations to the GenAI-assisted academic writing context were subsequently reviewed by three experts in the relevant field for semantic equivalence, clarity, and contextual appropriateness. All three experts agreed that the final items adequately reflected the intended constructs and retained the meaning of the original measures.

In addition, an open-ended question was included at each measurement point to elicit learners’ reflections on their experiences with the GenAI-enhanced academic writing course. Participants were invited to describe their perceptions, feelings, challenges, and learning experiences when using GenAI to support their academic writing. These qualitative responses were used to contextualise the quantitative findings and provide further insights into the dynamic development of learners’ WTW, ENY, and ANX in AI-IDLE.

#### 3.2.2. GenAI Chatbot

The GenAI tool used in this study was a customised chatbot developed on the Doubao Agent platform. Unlike general-purpose GenAI chatbots, the Agent was configured with task-specific prompts and instructions that corresponded to the weekly academic writing tasks in the EAP course. Rather than providing generic responses, it was designed to offer contextualised support for idea generation, argument development, academic organisation, language refinement, and revision throughout the writing process.

The chatbot enabled extended, multi-turn interactions, allowing learners to iteratively discuss writing topics, seek clarification, refine drafts, and explore alternative expressions. This conversational design supported continuous dialogue during writing rather than isolated question-and-answer exchanges.

The system also supported multimodal interaction through text, voice, and image inputs and was accessible across smartphones, tablets, and computers. Its cross-platform availability enabled learners to access the chatbot conveniently whenever writing support was needed.

### 3.3. Data Analysis

Data were analysed in RStudio (Version 2024.04.2+764). Following Peng and Liang’s (2025) intensive longitudinal design, descriptive statistics were calculated for WTW, ENY, and ANX at each of the 11 measurement occasions, and mean trajectories were plotted to describe their group-level temporal patterns. Individual WTW trajectories were also examined alongside the group-level trajectory to capture inter-individual variability that might be obscured by aggregate patterns.

CPA was then conducted to identify potential transitions in the development of WTW, ENY, and ANX. At the group level, CPA was applied separately to the mean trajectories of the three variables. CPA was implemented using the changepoint package in RStudio, with changes in the mean detected using the Pruned Exact Linear Time (PELT) algorithm and the modified Bayesian information criterion (MBIC) penalty. A minimum segment length of three measurement occasions was specified to reduce the identification of isolated short-term fluctuations as separate change segments. Change points were identified through penalised optimisation rather than conventional null-hypothesis significance testing. Specifically, PELT selected the segmentation that minimised the MBIC-penalised cost, such that additional change points were retained only when the improvement in model fit was sufficient to offset the corresponding penalty. At the individual level, the same procedure was additionally applied to each learner’s WTW trajectory to examine the occurrence and distribution of potential developmental transitions across participants. Detected change points were interpreted as the endpoints of preceding segments and therefore as potential transition boundaries between the identified occasion and the subsequent occasion. Given the limited number of measurement occasions, particularly the 11 observations available for each group-level series, detected change points were treated as indicators of potential transitions. Conversely, the absence of a detected change point was not interpreted as evidence that change was necessarily gradual or that abrupt changes were absent. To further examine the robustness of the group-level WTW change point, sensitivity analyses were conducted by varying key analytical specifications. Specifically, the primary PELT model with the MBIC penalty and a minimum segment length of three occasions was compared with (a) a PELT model using a minimum segment length of two occasions, (b) a PELT model using the SIC penalty, and (c) a Binary Segmentation model using the MBIC penalty. These analyses were used to assess whether the presence and approximate timing of the detected transition depended on the specific algorithm or parameterisation.

To examine temporal variation in the within-person associations between WTW and the two affective variables, MWC analysis was conducted using a three-occasion centred sliding window. For each participant separately, Pearson correlations between WTW and ENY and between WTW and ANX were calculated across successive three-occasion windows (T1–T3, T2–T4, …, T9–T11), with each correlation assigned to the central measurement occasion (T2–T10). The resulting coefficients therefore represented correlations across repeated observations within individual participants, rather than correlations across participants at a given measurement occasion. The individual-level correlation coefficients were then averaged across participants at each window to characterise the overall temporal pattern. Given that each individual correlation was estimated from only three consecutive observations, the MWC results were treated as exploratory and descriptive indicators of temporal variation in within-person covariation rather than as inferential evidence of structural reorganisation.

To assess potential selection associated with longitudinal participation, participant retention was examined across the 11 measurement occasions. In addition, the first available WTW, ENY, and ANX records of participants included in the primary analytic sample (n = 26) were compared with those of the remaining respondents (n = 85). Welch’s independent-samples *t*-tests were conducted. Mean differences and their 95% confidence intervals were reported, along with Hedges’ g as a standardised effect-size estimate. Holm adjustment was applied across the three comparisons. These analyses were used to assess observable differences in initial measured characteristics and were not interpreted as establishing equivalence between the groups or as ruling out selective attrition associated with subsequent participation.

Qualitative analysis was conducted using a structured codebook thematic approach, drawing on the general principles of thematic analysis (Braun & Clarke, 2006, 2021). First, the reflections were analysed inductively to identify recurring patterns across the dataset, from which descriptive codes were developed and progressively refined into a structured codebook. The coding unit was the student–occasion combination. When a participant submitted multiple reflections within the same measurement occasion, these responses were considered together, and each theme was counted only once for that participant at that occasion. Themes were not mutually exclusive.

For descriptive temporal comparison, the coded data were subsequently organised into four non-overlapping analytical periods: T1–T2 (initial engagement), T3–T6 (developing engagement), T7–T9 (later engagement), and T10–T11 (final period). These periods broadly reflected learners’ progression from initial exposure to GenAI, through accumulating experience alongside progressively structured academic writing activities, to the later and final stages of engagement. They were used as a descriptive framework for temporal comparison rather than as independently derived qualitative developmental stages. The qualitative findings were considered alongside the CPA and MWC results to illustrate and contextualise learners’ experiences rather than to independently confirm or validate the quantitative patterns.

Two researchers independently coded the qualitative data using the structured codebook. Inter-coder agreement exceeded 90%, and discrepancies were discussed until consensus was reached. All reflections were analysed in their original language. Chinese quotations selected for reporting were translated into English and subsequently reviewed by both researchers for semantic accuracy and consistency with the original text. Only translations agreed upon by both researchers were included in the manuscript.

## 4. Results

Before the longitudinal trajectories were examined, the first available WTW, ENY, and ANX records were compared between participants in the primary analytic sample (n = 26) and the remaining respondents (n = 85). No statistically significant differences were found (all unadjusted *p*s ≥ .550; all Holm-adjusted *p*s = 1.000). Standardised differences were small (|Hedges’g| ≤ 0.114), and all 95% confidence intervals for the mean differences included zero. These findings do not establish equivalence between the groups or rule out selection associated with subsequent participation.

### 4.1. Dynamic Development of WTW, ENY, and ANX

**Figure 1:**
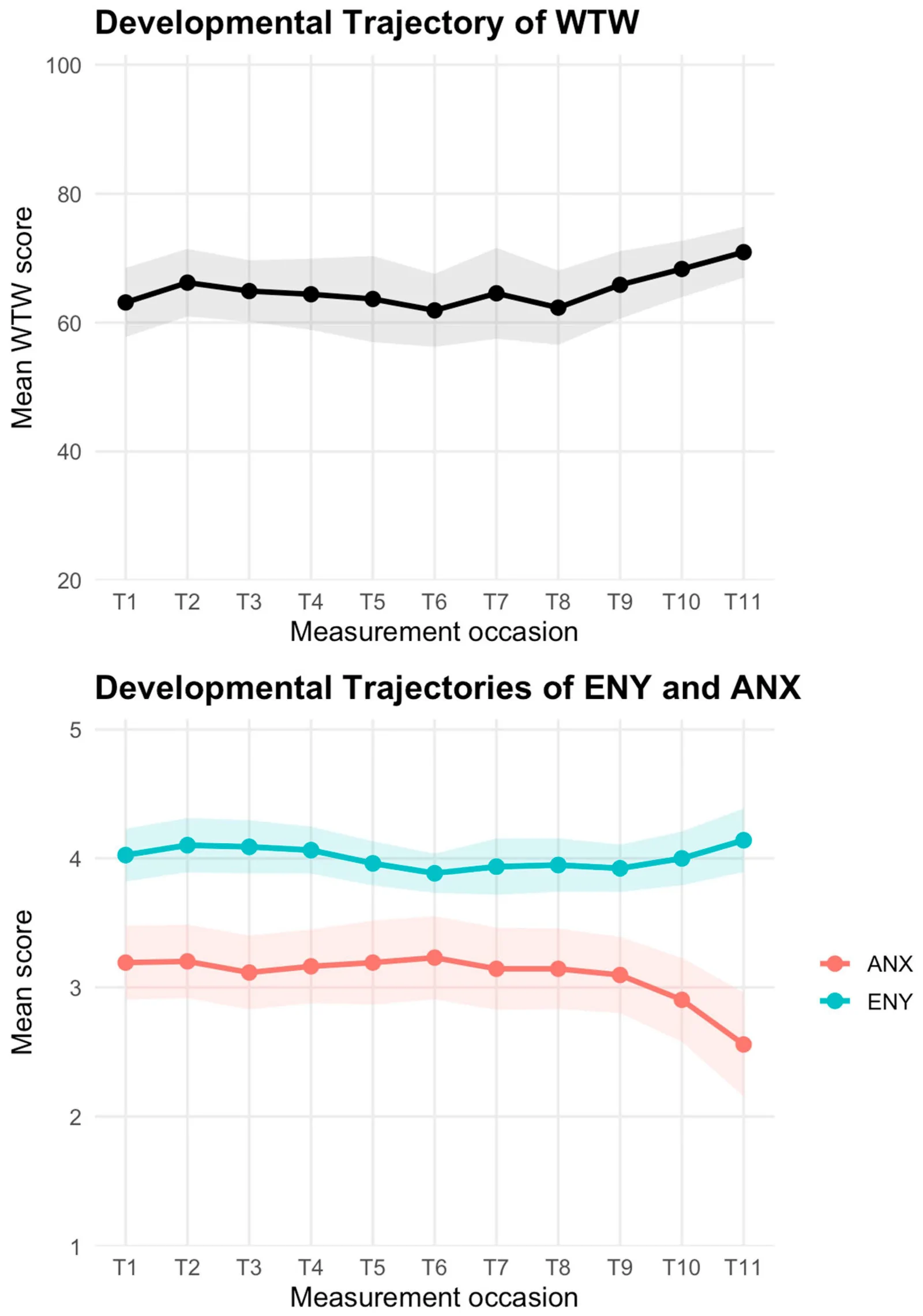
Developmental trajectories of WTW, ENY, and ANX across measurement points.

Table 1 presents the descriptive statistics for WTW, ENY, and ANX across the 11 measurement occasions. As shown in Table 1 and Figure 1, WTW exhibited a nonlinear developmental pattern, characterised by fluctuations across the earlier and middle measurement occasions, followed by a sustained increase after T8. The mean WTW score reached its lowest observed level at T6 (M = 61.88, SD = 14.02) and its highest at T11 (M = 70.92, SD = 9.78). ENY showed comparatively modest variation across the 11 occasions, declining slightly during the middle measurement occasions before increasing toward the end of the study. In contrast, ANX remained relatively stable from T1 to T8 and then declined during the later measurement occasions, from T9 (M = 3.10, SD = 0.73) to T11 (M = 2.56, SD = 1.00). Overall, the descriptive trajectories indicate temporal fluctuation in WTW, comparatively modest variation in ENY, and a later decline in ANX. The potential transitions underlying these observed patterns were subsequently examined using CPA.

### 4.2. CPA of WTW, ENY, and ANX

**Figure 2:**
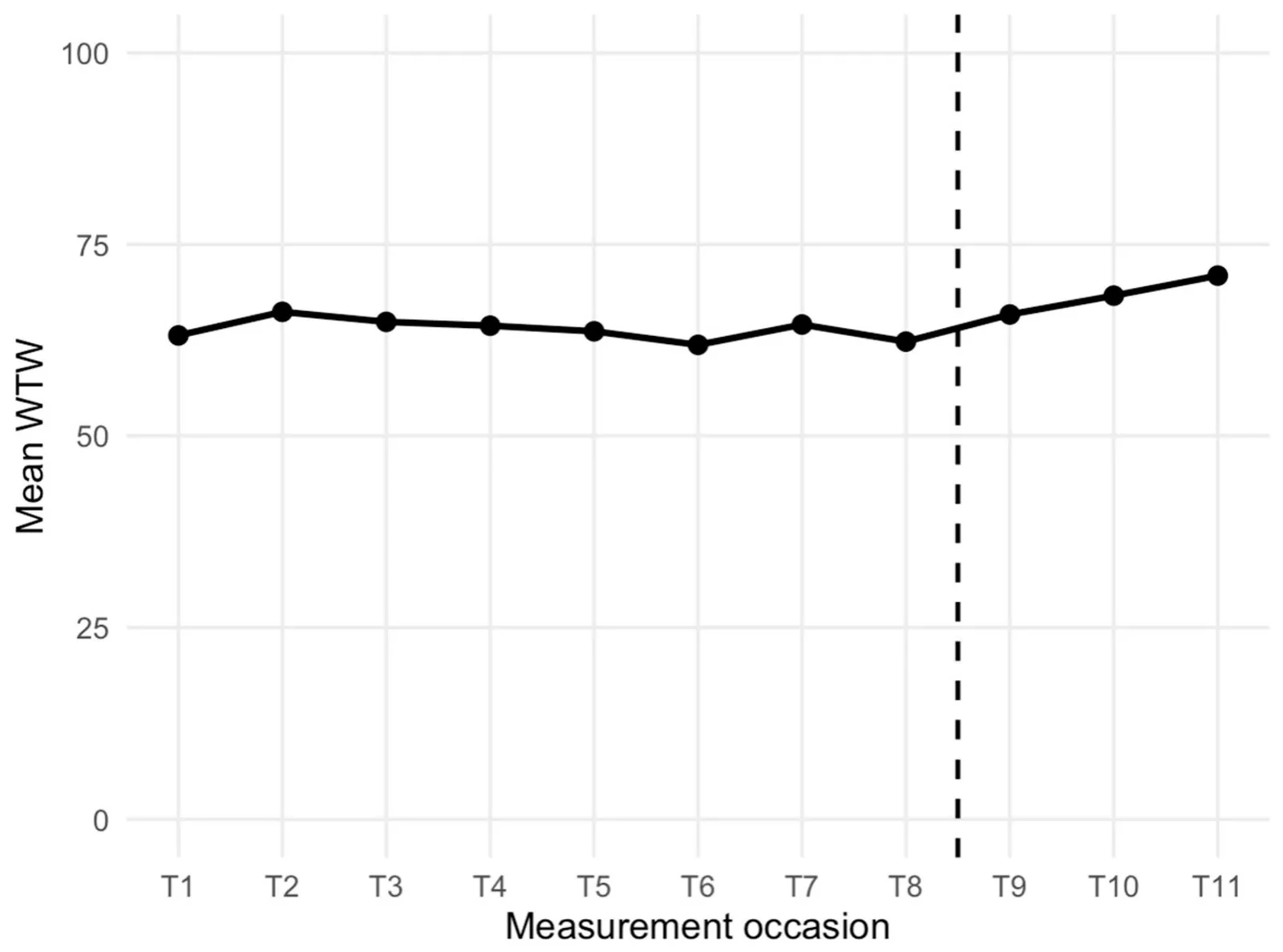
CPA of the group-level WTW trajectory. Note: The dashed vertical line indicates the detected change-point boundary between T8 and T9.

At the group level, CPA identified a single change-point boundary in WTW between T8 and T9 (Figure 2). Before this boundary, mean WTW fluctuated across the measurement occasions, reaching its lowest observed value at T6 (M = 61.88) and remaining relatively low at T8 (M = 62.31). Following the detected boundary, WTW increased consistently across the final three measurement occasions, from 65.85 at T9 to 68.31 at T10 and 70.92 at T11. The detected change point therefore indicates a potential late-stage transition in the group-level WTW trajectory.

Sensitivity analyses provided further support for a late-series change point in WTW, although its precise location depended on the minimum segment length. The T8–T9 boundary was retained when the penalty was changed from MBIC to SIC and when PELT was replaced by Binary Segmentation. Reducing the minimum segment length from three to two occasions shifted the detected boundary to T9–T10. Thus, a late-series change point in WTW was detected across all four specifications, with the estimated boundary located either between T8 and T9 or between T9 and T10.

**Figure 3:**
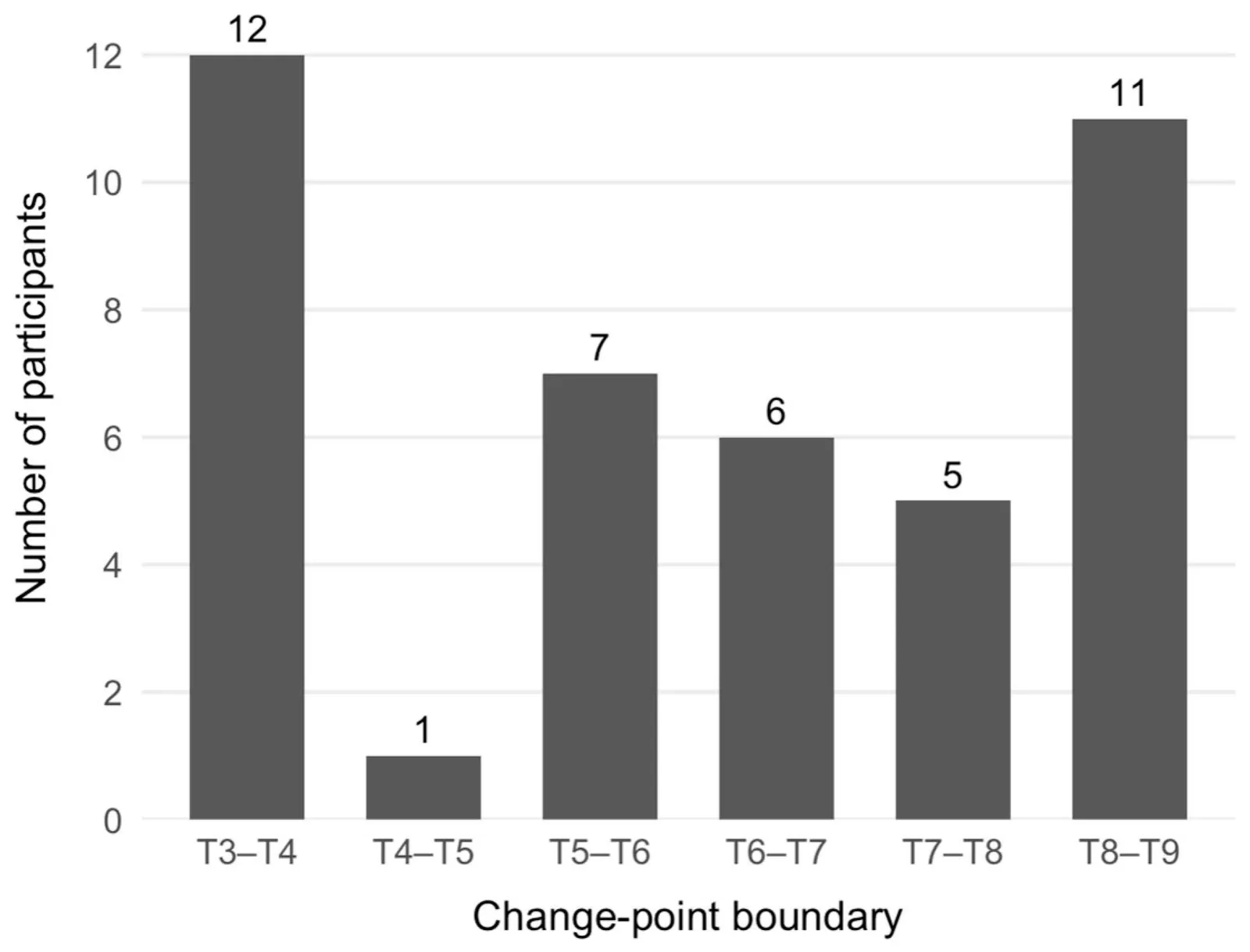
Distribution of individual-level WTW change points across measurement occasions. Note: Participants with two detected change points contribute to two bars.

Individual-level CPA revealed considerable heterogeneity in the timing of WTW transitions (Figure 3). Change points were detected for 25 of the 26 participants (96.2%), whereas one participant showed no detectable change point. Eight participants had one detected change point and 17 had two. The detected change-point boundaries were distributed from T3–T4 to T8–T9, with the largest concentrations occurring at T3–T4 (n = 12) and T8–T9 (n = 11). Notably, 11 participants (42.3%) exhibited an individual-level change point at the same T8–T9 boundary identified in the group-level analysis. Thus, although the individual-level results provide some support for the late-stage transition observed in the aggregate WTW trajectory, the distribution of change points across multiple occasions indicates substantial inter-individual variability in the timing of WTW development.

No change points were detected in the group-level trajectories of ENY or ANX. ENY showed relatively modest variation across the 11 measurement occasions, whereas ANX remained comparatively stable during the earlier and middle occasions before declining toward the end of the study, from 3.10 at T9 to 2.56 at T11. Given the limited number of observations in the group-level time series, however, the absence of detected change points for ENY and ANX should not be interpreted as evidence that changes in these variables were necessarily gradual.

### 4.3. Dynamic Relationships Between WTW and Affective Variables

**Figure 4:**
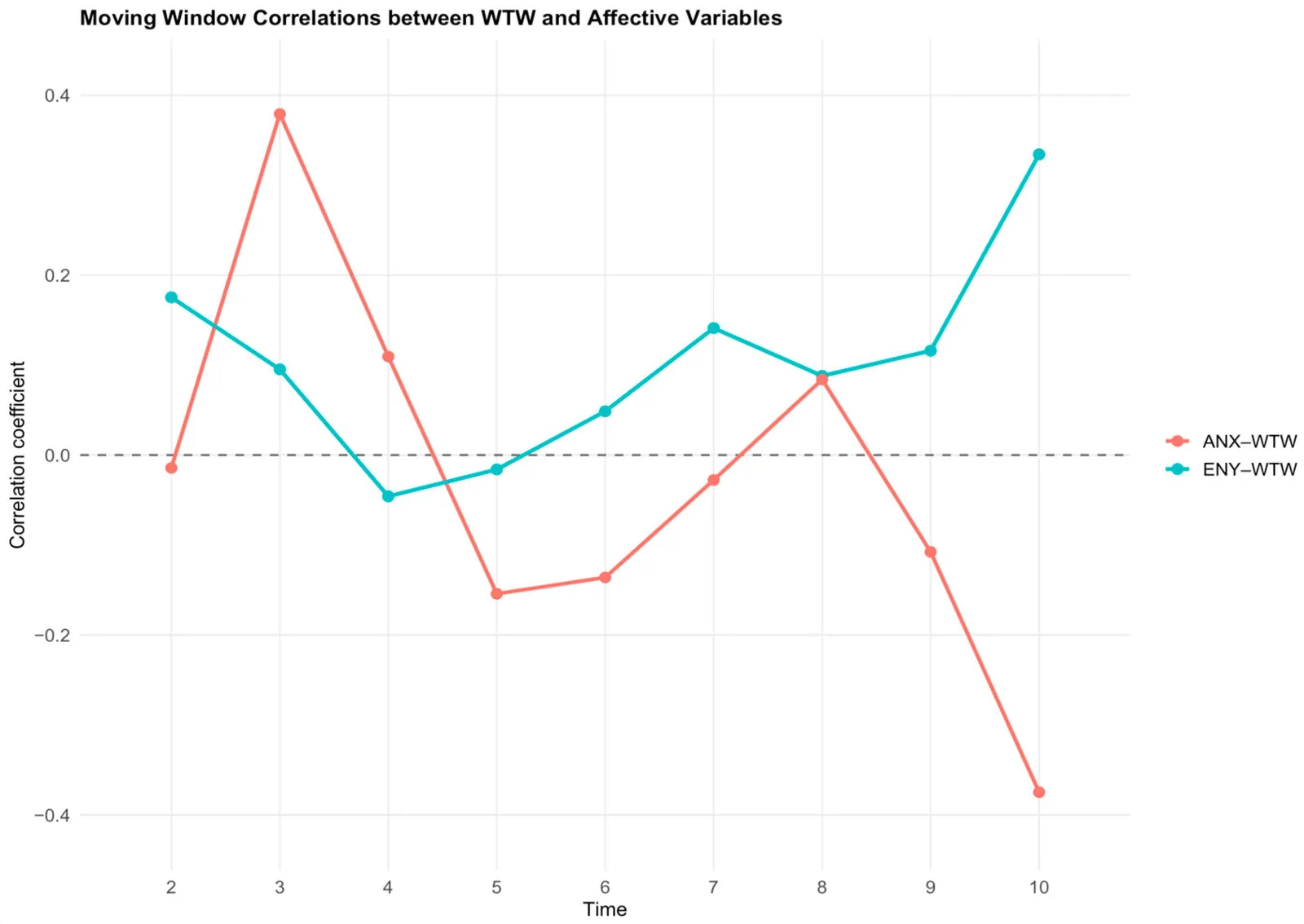
MWC between WTW and affective variables across measurement occasions. Note: Correlations were calculated separately within each participant using three-occasion centred windows and subsequently averaged across participants at each window.

Figure 4 presents the MWC between WTW and the two affective variables across the measurement occasions. The ENY–WTW correlation was generally positive but fluctuated over time, becoming slightly negative at T4 and T5 before becoming more positive during the later measurement occasions and reaching its highest observed value at T10 (mean r = 0.34). In contrast, the ANX–WTW correlation showed greater variability in both direction and magnitude, shifting between positive and negative values across the measurement occasions. The highest positive correlation was observed at T3 (mean r = 0.38), whereas the strongest negative correlation was observed at T10 (mean r = −0.38).

Descriptively, these patterns indicate temporal variation in the within-person covariation of WTW with ENY and ANX. During the later measurement occasions, the ENY–WTW correlations became more positive, whereas the ANX–WTW correlations became more negative. These patterns occurred during the same later part of the observation period in which WTW increased and the CPA identified a potential late-stage transition. Given that each participant-specific MWC was estimated from only three consecutive observations, however, the coefficients are inherently unstable and are therefore interpreted as exploratory and descriptive indicators of temporal variation in within-person covariation rather than as inferential evidence of structural reorganisation.

### 4.4. Students’ Perceptions of AI-IDLE Writing

#### 4.4.1. Novelty-Driven Orientation (T1–T2)

During T1–T2, 25 students described concrete writing support or knowledge gains, while two explicitly expressed novelty or interest. P09 described the experience as *“quite novel and interesting”* (T1), while P01 found it *“efficient and practical”* and able to address *“key problems in my writing”* (T1). Students discussed assistance with organising papers, locating literature, improving academic expression, and checking citation formats. These accounts placed particular emphasis on the immediate usefulness of the chatbot for making writing tasks more manageable.

Early engagement also included reservations about the tool’s limitations, with 19 students raising concerns about the depth or specificity of its guidance. In the same T1 reflection, P01 reported that its suggestions sometimes *“lacked depth”* and that personalised guidance was insufficiently precise. Initial enthusiasm thus coexisted with critical evaluation. The early phase is therefore best characterised as an orientation towards exploring the tool’s usefulness, with novelty contributing to some students’ interest.

#### 4.4.2. Rising Task Complexity and Perceived Depth Limitations (T3–T6)

During T3–T6, reflections increasingly addressed more demanding aspects of academic writing, including organising arguments, identifying research gaps, and developing introductions and literature reviews. Students described learning how to connect sections and explain the rationale for their research. Twenty-three students raised concerns about the depth or specificity of the chatbot’s guidance, and 23 identified limitations in personalisation or task fit. These overlapping themes concerned whether general frameworks could provide sufficiently detailed guidance for particular research topics.

P15 reported that the chatbot offered insufficient support for *“exploring the research background in depth and precisely articulating the significance of the study”* (T5). P19 identified a similar gap between diagnosis and actionable feedback: the chatbot could indicate that an introduction lacked a statement of research value but struggled to explain *“how to present that value in relation to the characteristics of the discipline”* (T5). These comments illustrate concerns about moving from structural guidance to substantive, context-sensitive argumentation.

Such concerns did not amount to a shared rejection of AI support. P10, for example, reported that the chatbot answered the questions accurately and added, *“I have not noticed any shortcomings”* (T5). Students thus differed in their assessments of how well the tool met their writing needs during this phase. They continued to value its organisational support, but their views differed on whether its guidance offered sufficient disciplinary depth and individualised feedback.

#### 4.4.3. Strategic Integration (T7–T9)

During T7–T9, 15 students described complementary roles for AI and human interlocutors. Students who favoured a combined approach described AI as useful for obtaining an initial framework, organising information, and addressing routine writing questions. They valued peer discussion for developing interpretations and considering alternative perspectives.

P06 explained, *“I prefer to combine the two: AI can quickly provide a framework, while peers can generate ideas about the details”* (T9). P07 made the task distinction more explicit: *“For basic organisation and framework-building, I prioritise AI tools; for in-depth discussion and the exchange of ideas, I prioritise discussion with peers”* (T9). These accounts indicate a selective approach in which students matched sources of support to particular writing demands.

Preferences nevertheless varied. Some students favoured AI because of its immediate availability, whereas others preferred the contextual understanding offered by face-to-face discussion. Strategic integration was therefore one identifiable pattern within this phase rather than a practice shared by all participants. Where it occurred, it involved recognising the complementary contributions of rapid AI assistance and human discussion.

#### 4.4.4. Routinisation and More Discerning Critique (T10–T11)

During the final phase, 17 students explicitly reported continued out-of-class or need-based use, including 10 who described frequent use or recurring usage situations. These explicit reports occurred at T11 and included activities such as organising literature, refining wording, and checking references. P19 stated, *“I have continued to use it frequently after class. It has become a standard tool for my academic writing and research”* (T11). P05 described a specific pattern of use: *“When I get stuck while writing, I ask it how to adjust the wording of the acknowledgements or check the reference-list format. It is much faster than looking through materials myself”* (T11). These reflections illustrate how the tool had become part of some students’ writing routines.

Continued use could coexist with detailed criticism. Seven students raised specific concerns about the accuracy or authenticity of AI-generated content during T10–T11. P01 noted that explanations of citation-format details were occasionally *“inconsistent with the official manual”* (T10), while P12 cautioned that the chatbot could *“fabricate reference information”* (T11). Such comments identified specific aspects of AI-generated output that required verification. For these students, recognising the practical value of the chatbot was compatible with questioning the reliability of the assistance it provided.

Routinisation was not universal. P25 reported, *“The overall experience was good, but I did not continue using it after class”* (T11). Positive evaluations of the tool therefore did not necessarily translate into sustained independent use. Likewise, concerns about accuracy had appeared earlier, so later criticism should not be interpreted as the first emergence of critical awareness. The final reflections instead show varied combinations of continued use, selective reliance, and scrutiny of AI-generated content.

Across the four phases, students’ accounts highlighted both the utility and the limitations of GenAI writing support. Later reflections included more explicit descriptions of human–AI complementarity and out-of-class use, while concerns about depth, disciplinary fit, and reliability persisted. These patterns suggest differentiation in how students understood and used the chatbot, with substantial variation in their preferences and practices.

## 5. Discussion

The discussion follows the developmental logic of the four research questions. Section 5.1 and Section 5.2 interpret the trajectories and change-point patterns of WTW, ENY, and ANX, thereby addressing RQ1 and RQ2. Section 5.3 then examines how the relationships between WTW and the two affective variables varied over time, addressing RQ3. Learners’ reflections are woven throughout all three sections to contextualise these quantitative patterns, thereby addressing RQ4 and strengthening the integration of the mixed-methods findings across the discussion.

### 5.1. Nonlinear Development of WTW in Teacher-Supported AI-IDLE Writing

The findings indicate that WTW followed a nonlinear developmental trajectory across the 11 measurement occasions. After reaching a mean of 66.19 at T2, WTW generally declined during the middle measurement occasions, falling to 61.88 at T6, before increasing during the later part of the study and reaching its highest observed level of 70.92 at T11. The primary CPA identified a potential change point at the T8–T9 boundary. Sensitivity analyses consistently identified a late-stage change point in WTW, although its precise location showed some sensitivity to the minimum segment length: reducing this requirement from three to two occasions shifted the detected boundary from T8–T9 to T9–T10. Individual-level CPA further revealed substantial heterogeneity in the timing of detected change points across participants. Although at least one change point was detected for 25 of the 26 participants, only 11 participants (42.3%) exhibited a change point at the T8–T9 boundary identified in the primary group-level analysis. The remaining change points were distributed across several earlier boundaries, indicating considerable inter-individual variation in the timing of WTW development. Such heterogeneity is consistent with the CDST emphasis on developmental pathways that may differ across individuals in both timing and form (De Bot et al., 2007; Larsen-Freeman, 1997). Accordingly, the group-level change point is better understood as a feature of the aggregated trajectory than as a uniform developmental transition experienced by all learners.

From a CDST perspective, this nonlinear WTW trajectory is consistent with the view that motivational development may involve periods of relative stability, fluctuation, and more pronounced change rather than following a uniformly linear trajectory (De Bot et al., 2007; Larsen-Freeman, 1997). Recent CDST-informed studies have similarly shown that L2 emotional and motivational development may exhibit considerable variability and non-gradual change (Ranjbar et al., 2025; Q. Zhou, 2024). The present study extends this developmental perspective to WTW in teacher-supported AI-IDLE writing, a context in which formal instructional structure coexists with learner-directed out-of-class digital engagement. As recent IDLE research has suggested, teacher involvement does not necessarily eliminate learner agency; teachers may provide instructional structure and initial support while learners retain meaningful control over their subsequent out-of-class digital practices (Kusuma et al., 2024; Guo et al., 2026; Lee et al., 2026). The present findings indicate that WTW may remain dynamic even within such a teacher-supported context. However, the CPA results alone are insufficient to establish system-level reorganisation or the emergence of a stable attractor state. Moreover, the limited number of measurement occasions following the potential late-stage change point prevents conclusions about whether it represented a stable longer-term developmental pattern.

The mid-course decline in WTW further highlights the importance of situating AI-IDLE within its broader instructional context. Learners’ out-of-class GenAI engagement did not occur independently of formal learning requirements but developed alongside progressively structured EAP writing activities. The decline in WTW coincided with a period of increasing academic writing demands, while learners’ reflections during this period frequently identified limitations in the depth, specificity, and personalisation of the chatbot’s responses. This is broadly consistent with previous accounts suggesting that sustaining willingness across extended writing may become more difficult as writing demands accumulate (Boscolo & Gelati, 2019; Kaivanpanah et al., 2019; Yang & Zhang, 2023). This finding also illustrates the importance of distinguishing task initiation from digital engagement when conceptualising AI-IDLE writing. Academic writing activities may originate from coursework or other formal instructional requirements (Seviour, 2015), while learners may still retain agency over whether, when, and how to mobilise digital resources outside the classroom to support these activities (Guo et al., 2026; Lee et al., 2026).

The qualitative findings provide further context for these quantitative patterns. During the initial period (T1–T2), learners’ reflections frequently emphasised the writing support provided by GenAI, alongside some expressions of novelty and interest. During T3–T6, when WTW was generally lower, learners frequently identified limitations in the depth, specificity, and personalisation of the chatbot’s responses. Previous research has similarly reported concerns regarding generic, inaccurate, or insufficiently task-specific GenAI support (L. Sun et al., 2026; C. Wang et al., 2026; Zou et al., 2025). During the later period (T7–T9), learners increasingly articulated differentiated roles for AI and human support, suggesting a growing recognition that different sources of support were better suited to different aspects of academic writing. In the final period (T10–T11), some learners further described continued, need-based, or more routinised forms of GenAI use.

The later reflections are particularly informative for understanding how learner-directed engagement may develop within teacher-supported AI-IDLE. Some participants reported using GenAI for brainstorming, initial organisation, or language refinement while relying on peers or other human resources for conceptual development, disciplinary judgement, or critical evaluation. These accounts do not suggest that the chatbot itself became more effective; rather, they indicate that some learners developed a more differentiated understanding of its affordances and limitations and more selective strategies for integrating it into academic writing. Previous studies have raised concerns that extensive GenAI use may lead to learner dependence and constrain the development of independent writing strategies (Jeon et al., 2023; Liang et al., 2024; G. L. Liu et al., 2025). The reflections in the present study, however, indicate that learners’ ways of engaging with GenAI did not necessarily remain unchanged throughout the study. As their experience accumulated, some participants increasingly described selective, critical, and task-specific forms of GenAI use.

Importantly, however, this development occurred within teacher-supported AI-IDLE rather than in a context detached from instruction. The chatbot was initially introduced and demonstrated by the instructor, while learners’ subsequent out-of-class engagement developed alongside formal EAP instruction and progressively structured writing activities. The present design therefore cannot disentangle the respective contributions of task progression, accumulated GenAI experience, formal instruction, broader learner development, and other time-related influences. Consequently, the later increase in WTW cannot be attributed specifically to learners’ increasingly strategic use of GenAI, just as the mid-course decline cannot be attributed solely to increasing task complexity. A more appropriate interpretation is that the observed trajectory illustrates how learner-directed GenAI engagement may develop within and alongside formal instructional support. This finding further aligns with recent conceptualisations of IDLE in which teacher support and learner agency are not treated as mutually exclusive dimensions (Kusuma et al., 2024; Guo et al., 2026; Lee et al., 2026).

Viewed from a human-intelligence perspective, these later patterns may reflect the exercise of adaptive and metacognitive capacities in AI-mediated writing. Learners were not merely receiving AI-generated assistance; some increasingly evaluated its limitations, differentiated appropriate uses of human and artificial support, and adjusted their strategies accordingly. Human intelligence in this context is therefore reflected not in replacing AI-generated output with unaided human performance, but in the capacity to judge, regulate, and strategically coordinate interaction with AI.

### 5.2. Technology-Related Enjoyment and Anxiety as Dynamic Emotional Subsystems

Unlike WTW, neither ENY nor ANX exhibited a detectable change point under the primary CPA specification, although their descriptive trajectories differed. ENY remained relatively stable across the 11 measurement occasions, whereas ANX generally declined, with lower mean levels observed particularly during the final measurement occasions. Importantly, the absence of a detected change point does not imply that these emotions changed only gradually, nor does it demonstrate that abrupt changes were absent. More precisely, no change point met the detection criterion under the present data and analytical specification.

The different trajectories of ENY and ANX are consistent with the view that technology-related enjoyment and anxiety should be regarded as related but distinct affective processes rather than opposite ends of a single dimension (De Bot et al., 2007). In the early reflections, learners described the chatbot as “novel” or “interesting”, whereas later reflections placed greater emphasis on its accessibility, convenience, and role in their writing practices. Although these reflections suggest that the sources of positive experiences may have changed over time, mean ENY remained relatively stable overall. This pattern is consistent with technology-acceptance research suggesting that positive technology-related experiences may arise not only from novelty but also from factors such as perceived usefulness and accessibility (Venkatesh et al., 2012; Zou et al., 2025). It also aligns with previous research suggesting that enjoyment may play an important role in sustaining engagement in AI-supported language learning (Huang & Zou, 2024).

ANX followed a different descriptive pattern, generally declining across the observation period. Notably, learners continued to identify limitations in GenAI-generated content throughout the study, including generic responses, inaccuracies, and citation-related problems. The qualitative findings suggest that the ways in which learners articulated these concerns may have changed over time. During the middle analytical period, reflections more often raised relatively broad concerns about insufficient depth, specificity, or personalisation, whereas some later reflections identified more specific problems concerning the accuracy or appropriateness of AI-generated content. One possible interpretation is that, as their experience accumulated, some learners developed clearer expectations about the limitations they might encounter and how they could respond to them. This interpretation is consistent with conceptualisations of technology-related anxiety that emphasise uncertainty, system reliability, and unpredictability (C. Wang et al., 2026; Zou et al., 2025). This interpretation, however, should be considered in light of the broader learning context. Learners did not engage with the technology in isolation; their out-of-class GenAI engagement developed concurrently with formal EAP instruction, progressively structured writing activities, and accumulating academic writing experience. This contextual interdependence is particularly important in teacher-supported AI-IDLE, because out-of-class digital engagement may remain connected to formal instructional contexts even when learners retain substantial agency over their technology use (Kusuma et al., 2024; Guo et al., 2026; Lee et al., 2026).

Overall, the different trajectories of ENY and ANX are consistent with the CDST perspective that different affective experiences may develop in different ways over time rather than changing synchronously or simply in opposite directions (Dewaele & Dewaele, 2017; De Bot et al., 2007). In the present study, relatively stable enjoyment coexisted with anxiety that declined particularly during the final measurement occasions, while learners simultaneously progressed through formal academic writing activities and accumulated experience with GenAI. The present findings therefore extend a dynamic perspective on L2 emotion to technology-related affect in teacher-supported AI-IDLE writing, while also highlighting an important point: the development of technology-related emotions is difficult to separate from the instructional context and broader learner development within which it occurs.

### 5.3. Temporal Variation in the ENY–WTW and ANX–WTW Relationships

The MWC analysis indicated temporal variation in the within-person covariation of WTW with ENY and ANX across the observation period. The ENY–WTW correlations were generally positive but fluctuated across windows, becoming more positive during the later measurement occasions and reaching their highest observed mean value at T10 (r = 0.34). The ANX–WTW correlations showed greater variation in both direction and magnitude, with the strongest positive correlation observed at T3 (r = 0.38) and the strongest negative correlation at T10 (r = −0.38). At a descriptive level, these findings indicate that the patterns of covariation between WTW and the two affective variables were not constant throughout the study.

These findings are consistent with the relational emphasis of CDST. Rather than focusing solely on changes within individual variables, CDST also draws attention to how relationships among motivational and affective processes may vary over time (De Bot et al., 2007; Larsen-Freeman, 1997). However, each participant-level MWC coefficient was calculated from only three consecutive measurement occasions and is therefore inherently unstable. Accordingly, these correlations should be regarded as exploratory and descriptive rather than as evidence of structural reorganisation in the underlying relationships among WTW, ENY, and ANX.

The later ENY–WTW pattern occurred during approximately the same period in which some learners described more established and selective ways of using GenAI in their reflections. Early positive evaluations tended to focus on novelty or immediate usefulness, whereas later accounts placed greater emphasis on accessibility, efficiency, and task-specific use. Given that WTW involves sustained engagement in writing processes such as planning, drafting, integrating sources, and revising rather than a single momentary act (Zhang & Zhang, 2024; Zou et al., 2025), accumulated experience with GenAI may be related to how enjoyment and willingness to write covary over an extended period of writing.

The ANX–WTW pattern was less consistent across the observation period. Positive correlations were observed in some earlier windows, whereas more pronounced negative correlations emerged in some later windows. This variability is consistent with recent research suggesting that active engagement with GenAI can coexist with anxiety rather than necessarily implying that anxiety is displaced by positive experiences (F. Sun et al., 2026). Learners’ reflections likewise suggest that the nature of their concerns may have changed as their experience accumulated, shifting from relatively broad concerns about the usefulness and appropriateness of AI-generated support towards more specific evaluations of its limitations. This provides one possible contextual interpretation of the changing ANX–WTW pattern.

Viewed within the broader conceptual framework of the study, these findings illustrate the dynamic characteristics of teacher-supported AI-IDLE embedded within formal EAP instruction. In this context, learner agency does not depend on the absence of formal instruction; rather, it is reflected in learners’ decisions about whether, when, how, and for what purposes to integrate GenAI into writing activities that are themselves formally structured within the curriculum (Guo et al., 2026; Lee et al., 2026). This further extends the emerging recognition that IDLE can coexist with formal instructional support rather than being defined solely by its separation from formal learning (Kusuma et al., 2024; Guo et al., 2026; Lee et al., 2026). The developmental trajectories and time-varying patterns of covariation observed in this study therefore emerged at the intersection of formal writing instruction and learner-directed out-of-class GenAI engagement. Overall, the findings suggest that WTW may develop nonlinearly, while its covariation with ENY and ANX may also exhibit exploratory temporal variation within a complex context in which task progression, accumulated GenAI experience, formal instructional support, and broader learner development unfold concurrently.

## 6. Conclusions, Implications and Limitations

This study adopted a CDST perspective to examine the longitudinal development of WTW, ENY, and ANX in teacher-supported AI-IDLE embedded within formal EAP instruction. Across 11 measurement occasions, WTW followed a nonlinear trajectory, declining during the middle period before increasing later. The primary CPA identified a potential late-stage change at T8–T9, although its precise location was somewhat sensitive to the minimum segment length, while individual-level CPA revealed substantial heterogeneity in the timing of WTW change points. In contrast, ENY remained relatively stable, whereas ANX generally declined. MWC analysis further indicated temporal variation in the within-person covariation of WTW with ENY and ANX. Together, these findings suggest that motivational and affective experiences in GenAI-supported writing may develop dynamically rather than following uniform linear trajectories. Viewed from a human-intelligence perspective, learners’ increasingly selective and critical engagement with GenAI further illustrates the adaptive, evaluative, and metacognitive capacities involved in navigating AI-mediated learning. These capacities were inferred from learners’ reported practices rather than directly measured as intelligence constructs; accordingly, the findings indicate how human intellectual capacities may be exercised in AI-mediated learning rather than demonstrating an increase in human intelligence.

Theoretically, this study extends a CDST perspective on L2 motivation and emotion to GenAI-assisted academic writing while contributing to the conceptualisation of AI-IDLE. Rather than demonstrating a uniform system-level reorganisation, the findings highlight nonlinear development, temporal variation, and substantial inter-individual heterogeneity. They also show that learner agency can coexist with formal instructional support. Although the writing activities were progressively structured and the chatbot was introduced and demonstrated by the instructor, learners retained discretion over whether, when, how, and for what purposes to use GenAI outside class. The observed dynamics therefore emerged at the intersection of formal writing instruction and learner-directed out-of-class GenAI engagement.

Pedagogically, the findings suggest that teachers should not assume that learners’ willingness to engage in GenAI-supported writing will increase steadily over time. Instead, teachers can support learners in developing more selective and strategic uses of GenAI and in establishing an appropriate division of labour among GenAI, peers, and teachers. Learners’ recurring concerns about generic, inaccurate, or insufficiently task-specific AI-generated content also highlight the importance of developing critical AI literacy, particularly the ability to evaluate, verify, and selectively use AI-generated information.

Several limitations should be acknowledged. First, the longitudinal analyses were based on 26 complete cases from a single institution. Although no clear initial differences in WTW, ENY, or ANX were found between retained participants and the remaining respondents, this does not establish equivalence or rule out selective attrition. The reported trajectories may therefore partly characterise learners who were particularly persistent in sustained participation rather than the broader course population. Second, the study examined one customised GenAI chatbot within a specific 11-week EAP course, and T1 was collected after the initial introduction of the chatbot rather than as a pre-exposure baseline. Third, the single-item WTW measure and the limited number of measurement occasions constrain the precision of the longitudinal analyses. In particular, the CPA indicates a potential late-stage change whose precise location was somewhat parameter-sensitive, while the three-occasion MWC coefficients should be interpreted as exploratory and descriptive. Finally, task progression, formal instruction, learner development, time, and accumulated GenAI experience unfolded concurrently and cannot be disentangled within the present design. The qualitative findings similarly contextualise rather than independently confirm or explain the quantitative patterns. Future research could employ larger and more diverse samples, denser longitudinal measurements, comparison conditions, and different GenAI tools to examine these developmental patterns more robustly.

## Figures and Tables

**Table 1:** Descriptive statistics for WTW, ENY, and ANX.

Time	WTW M (SD)	ENY M (SD)	ANX M (SD)
1	63.12 (13.28)	4.03 (0.51)	3.19 (0.71)
2	66.19 (12.99)	4.10 (0.52)	3.20 (0.71)
3	64.88 (11.83)	4.09 (0.51)	3.12 (0.71)
4	64.38 (13.68)	4.06 (0.45)	3.16 (0.71)
5	63.65 (16.57)	3.96 (0.43)	3.19 (0.81)
6	61.88 (14.02)	3.88 (0.38)	3.23 (0.80)
7	64.54 (17.45)	3.94 (0.54)	3.14 (0.79)
8	62.31 (14.27)	3.95 (0.51)	3.14 (0.77)
9	65.85 (12.97)	3.92 (0.46)	3.10 (0.73)
10	68.31 (10.82)	4.00 (0.52)	2.90 (0.80)
11	70.92 (9.78)	4.14 (0.61)	2.56 (1.00)

## Data Availability

The data supporting the findings of this study are available from the corresponding authors upon reasonable request.
